# Toll-like receptor 4 contributes to retinal ischemia/reperfusion injury

**Published:** 2010-09-30

**Authors:** Galina Dvoriantchikova, David J. Barakat, Eleut Hernandez, Valery I. Shestopalov, Dmitry Ivanov

**Affiliations:** 1 Bascom Palmer Eye Institute, Department of Ophthalmology, University of Miami Miller School of Medicine, Miami, FL; 2 Department of Cell Biology and Anatomy, University of Miami Miller School of Medicine, Miami, FL; 3 Vavilov Institute of General Genetics RAS, Moscow, Russian Federation

## Abstract

**Purpose:**

We investigated whether retinal ischemia and inflammation produced by raising the intraocular pressure above normal systolic levels differs in mice that lack a functional toll-like receptor 4 (Tlr4) signaling pathway.

**Methods:**

In this work we used the murine strain B6.B10ScN-Tlr4^lps-del^/JthJ, which does not express functional Tlr4. C57BL/6J was considered as the control. We induced retinal ischemia by unilateral elevation of intraocular pressure for 1 h by direct corneal cannulation. The changes in expression of proinflammatory genes 24 h postreperfusion were assessed by quantitative PCR. Corresponding changes in protein abundances were analyzed by western blot and immunohistochemistry. Cell death was evaluated by direct counting of neurons in the ganglion cell layer of flat-mounted retinas seven days postreperfusion.

**Results:**

We showed that Tlr4-deficient mice display significantly reduced expression of proinflammatory genes, including *RelA,* tumor necrosis factor *(Thf),* interleukin 6 *(Il6),* chemokine *(C-C motif)* ligand 2 *(Ccl2),* chemokine *(C-C motif)* ligand 5 *(Ccl5),* chemokine *(C-X-C motif)* ligand 10 *(Cxcl10), Cybb,* nitric oxide synthase 2 *(Nos2),* and intercellular adhesion molecule 1 *(Icam1)* 24 h after reperfusion. The mice that lacked Tlr4 showed significantly increased survival of neurons in the ganglion cell layer following ischemic injury, as compared to wild-type controls.

**Conclusions:**

Our results indicate that Tlr4 signaling is involved in retinal damage and inflammation triggered by ischemic injury.

## Introduction

Retinal ischemia is a common clinical entity and, due to relatively ineffective treatment, remains a common cause of visual impairment and blindness [1]. Ischemia has been proposed as a facet of anterior ischemic optic neuropathy, retinal and choroidal vessel occlusions, glaucoma, diabetic retinopathy, retinopathy of prematurity, and traumatic optic neuropathy [1–5]. Thus, increased understanding of the events involved in ischemic neuronal injury can provide us with clinically effective treatments for many retinal diseases.

Toll-like receptors (TLRs) have been identified in the central nervous system (CNS) and are thought to play an important role in the CNS response to pathogens, as well as necrotic cells [6–8]. Recent studies using a permanent and longstanding focal cerebral ischemia model have shown that infarct size is reduced in Tlr4-deficient mice compared with wild-type (WT) mice [9,10]. Another observation linked Tlr4 deficiency with the reduced neuronal death and lowered levels of proinflammatory cytokine expression in the hippocampus in models of global cerebral ischemia/reperfusion (IR) and axotomy-induced degeneration [11–13]. Tlr4 expression and localization in the retina has been extensively studied and reported [14–20]. Because of many parallels in cell death mechanisms in brain and retinal ischemia, we hypothesized that Tlr4 signaling is involved in retinal damage and in inflammation triggered by ischemic injury.

## Methods

### Animals

All experiments and postsurgical care were performed in compliance with the NIH Guide for the Care and Use of Laboratory Animals and according to the University of Miami Institutional Animal Care and Use Committee approved protocols. Adult male B6.B10ScN-Tlr4^lps-del^/JthJ (stock number 007227) and C57BL/6J (stock number 000664) mice were obtained from the Jackson Laboratory (Bar Harbor, ME). The murine strain B6.B10ScN-Tlr4^lps-del^/JthJ does not express functional Tlr4. C57BL/6J was used as a control. Mice were housed under standard conditions of temperature and humidity, with a 12 h light/dark cycle and free access to food and water. All animals used in our experiments were three-month-old mice (six animals per group).

### Transient retinal ischemia

After anesthesia with intraperitoneal ketamine (80 mg/kg) and xylazine (16 mg/kg), pupils were dilated with 1% tropicamide–2.5% phenylephrine hydrochloride (NutraMax Products, Inc., Gloucester, MA), and corneal analgesia was achieved with 1 drop of 0.5% proparacaine HCl (Bausch and Lomb Pharmaceuticals, Tampa, FL). Retinal ischemia was induced for 60 min by introducing into the anterior chamber of the left eye a 33-gauge needle attached to a normal (0.9% NaCl) saline-filled reservoir raised above the animal to increase intraocular pressure (IOP) above cystolic blood pressure (IOP increased to 120 mmHg). The contralateral (right) eye was cannulated and maintained at normal IOP to serve as a normotensive control. Complete retinal ischemia, evidenced by a whitening of the anterior segment of the eye and blanching of the retinal arteries, was verified by microscopic examination. After needle removal, 1% atropine and 1% vetropolycin with hydrocortisone ointment (Fougera and Atlanta, Inc., Melville, NY) were applied to the conjunctival sac. Mice were sacrificed by CO_2_ inhalation under anesthesia.

### Immunohistochemistry for Neuronal Nuclei (NeuN) antibody in flat-mounted retinas

Eyes were enucleated upon euthanasia, incised at the ora serrata and immersion-fixed in a 4% paraformaldehyde solution (in phosphate buffered saline solution (PBS): 1.4 mM KH_2_PO_4_, 8 mM Na_2_HPO_4_, 140 mM NaCl, 2.7 mM KCl, pH 7.4) for 1 h, and the retinas were removed. The retinas were cryoprotected overnight in 30% sucrose followed by three freeze–thaw cycles, were rinsed 3×10 min in 0.1 M PBS, blocked by 5% donkey serum, 0.1% Triton X-100 in 0.1 M Tris buffer for 1 h, and incubated overnight with monoclonal fluorescein isothiocyanate–conjugated Neuronal Nuclei (NeuN) antibody (dilution 1:300; Chemicon, Billerica, MA). After 3×10 min rinsing in 0.1 M Tris buffer, retinas were flatmounted, coverslipped, and imaged using a Leica TSL AOBS SP5 confocal microscope (Leica Microsystems, Exton, PA).

### Counting of NeuN positive ganglion cell layer neurons

NeuN-positive neurons in the ganglion cell layer (GCL), including retinal ganglion cells and displaced amacrine cells, were imaged by confocal microscopy in flat-mounted retinas. To avoid topological irregularities, stacks of five serial images were collapsed to generate “maximum projections” (standard feature of the Leica LAS AF software), where all imaged cells appear in sharp focus. Individual retinas were sampled randomly to collect a total of 20 images located at the same eccentricity in the four retinal quadrants using a 20× objective lens. NeuN-positive neurons with a size range of 6–30 µm were counted semiautomatically using MetaMorph (Molecular Devices, Sunnyvale, CA) software, after image thresholding and the manual exclusion of artifacts. Cell loss in the ischemic retinas was calculated as percentile of the mean cell density in normotensive fellow control eyes.

### Real-time polymerase chain reaction analysis

Real-time PCR analysis was performed as described previously [21,22] using gene-specific primers (Table 1). Specifically, total RNA was extracted from retinas using Nanoprep (Stratagene, Santa Clara, CA), reverse transcribed with Superscript III polymerase (Invitrogen, Carlsbad, CA) to synthesize cDNA. Real-time PCR was performed in the Rotor-Gene 6000 Cycler (Corbett Research, Australia) using the SYBR GREEN PCR MasterMix (Qiagen, Valencia, CA). For each gene, relative expression was calculated by comparison with a standard curve, following normalization to the housekeeping gene β-actin (*Actb*) expression chosen as control.

**Table 1:** List of PCR primers

**Gene**	**Oligonucleotides**	
*Il1b*	Forward	GACCTTCCAGGATGAGGACA
	Reverse	AGGCCACAGGTATTTTGTCG
*Il6*	Forward	ATGGATGCTACCAAACTGGAT
	Reverse	TGAAGGACTCTGGCTTTGTCT
*Tnf*	Forward	CAAAATTCGAGTGACAAGCCTG
	Reverse	GAGATCCATGCCGTTGGC
*RelA*	Forward	GGCCTCATCCACATGAACTT
	Reverse	ATCGGATGTGAGAGGACAGG
*Ccl2*	Forward	AGGTCCCTGTCATGCTTCTG
	Reverse	ATTTGGTTCCGATCCAGGTT
*Ccl5*	Forward	AGCAGCAAGTGCTCCAATCT
	Reverse	ATTTCTTGGGTTTGCTGTGC
*Cxcl10*	Forward	GCTGCAACTGCATCCATATC
	Reverse	CACTGGGTAAAGGGGAGTGA
*Icam1*	Forward	TGGTGATGCTCAGGTATCCA
	Reverse	CACACTCTCCGGAAACGAAT
*Cybb*	Forward	GACTGCGGAGAGTTTGGAAG
	Reverse	ACTGTCCCACCTCCATCTTG
*Ncf1*	Forward	CGAGAAGAGTTCGGGAACAG
	Reverse	AGCCATCCAGGAGCTTATGA
*Ncf2*	Forward	CTACCTGGAGCCAGTTGAGC
	Reverse	AGCGCCAGCTTCTTAGACAC
*Gfap*	Forward	AGAAAGGTTGAATCGCTGGA
	Reverse	CGGCGATAGTCGTTAGCTTC
*Nos2*	Forward	CAGAGGACCCAGAGACAAGC
	Reverse	TGCTGAAACATTTCCTGTGC
*Actb*	Forward	CACCCTGTGCTGCTCACC
	Reverse	GCACGATTTCCCTCTCAG

Abbreviations:  *Il1β* represents interleukin 1β, *Il6* represents interleukin 6,  *Thf *represents tumor necrosis factor,  *RelA *represents transcriptional upregulation of the p65, *Ccl2 *represents chemokine (C-C motif) ligand 2, *Ccl 5* represents chemokine (C-C motif) ligand 5,  *Cxc10* represents chemokine (C-X-C motif) ligand 10, *Icam1 *represents intercellular adhesion molecule 1, *Ncf1* represents neutrophil cytosolic factor 1, *Ncf2 *represents neutrophil cytosolic factor 2, *Gfap* represents glial fibrillary acidic protein, *Nos2* represents nitric oxide synthase 2.

### Immunohistochemistry

Fixed retinas were sectioned to a thickness of 100 μm with a vibratome (Vibratome, St. Louis, MO) and immunostained using the protocol described earlier [21,22]. Briefly, sections were permeabilized with 0.3% Triton X-100 in 1xPBS for 45 min, rinsed in 1X PBS and blocked by 5% donkey serum, 2% BSA and 0.15% Tween-20 in 1X PBS for 1 h and incubated overnight with chemokine (C-C motif) ligand 2 (Ccl2; Sc-1784; Santa Cruz, Santa Cruz, CA) or interleukin 6 (Il6; AMC0864; Invitrogen, Carlsbad, CA) primary antibodies, followed by species-specific secondary fluorescent antibodies (AlexaFluor; Invitrogen). Control sections were incubated without primary antibodies. Imaging was performed with a Leica TSL AOBS SP5 confocal microscope (Leica Microsystems, Bannockburn, IL).

### Western blot analysis

Samples containing 20 µg of protein were loaded, and the proteins were size-separated in sodium dodecyl sulfate polyacrylamide gel (SDS–PAGE). Proteins were blotted onto a polyvinylidene difluoride (PVDF) membrane (Invitrogen) and incubated with phospho-p65 primary antibody. Proteins recognized by the antibody were revealed by the SuperSignal West Femto Maximum Sensitivity Substrate from Pierce according to instructions (Thermo Fisher Scientific, Rockford, IL). Briefly, luminol/enhancer and stable peroxide solutions were mixed at a 1:1 ratio to prepare the substrate working solution. PVDF membrane was incubated 5 min in working solution. Quantification of the protein bands were performed using the software Quantity One (Bio-Rad Laboratories, Hercules, CA). Data were normalized against β-actin.

### Statistical analysis

Statistical analysis of real-time PCR and cell density data was performed with one-way ANOVA followed by the Tukey test for multiple comparisons. In the case of single comparisons, the Student *t* test was applied. P values equal to or less than 0.05 were considered statistically significant.

## Results

### Inactivation of toll-like receptor 4 signaling promotes survival of retinal neurons following ischemic injury

**Figure 1:**
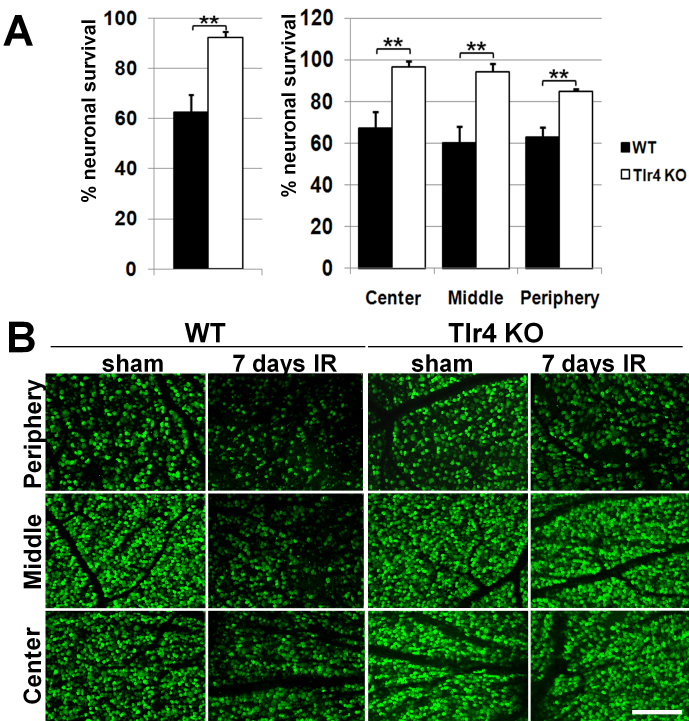
Toll-like receptor 4 deficiency results in neuroprotective effects in the ganglion cell layer of retinas after ischemia/reperfusion. **A**: The percent surviving of ganglion cell layer (GCL) neurons in the ischemia/reperfusion (IR) retinas of wild type (WT) and toll-like receptor 4 (Tlr4) knockout (KO) animals seven days after IR. The percent of Neuronal Nuclei (NeuN)-labeled neurons in regions of central, middle and peripheral retina were compared between sham operated and ischemic eyes of WT and Tlr4 KO animals seven days after IR (**p<0.01, n=6). **B**: Representative confocal images of NeuN-labeled GCLs (green) in flat-mounted controls and ischemic retinas seven days after reperfusion. Scale bar equal to 100 µm.

To investigate the role of Tlr4 in retinal ischemia, we took advantage of Tlr4-deficient mice. We induced unilateral retinal ischemia in WT and Tlr4 knockout (Tlr4 KO) mice by raising IOP above normal systolic levels and evaluating neuronal survival. High IOP-induced transient retinal IR is one of the most frequently used models to investigate molecular mechanisms contributing to neuronal ischemic injury [23]. This technique produces global ischemia by obstruction of both the retinal and uveal circulation, and causes retinal GCL pathology closely mimicking that observed in central retinal artery occlusion [1]. The IR-induced degeneration of neurons in the GCL is biphasic, with a primary degeneration occurring within 24 h after reperfusion and a secondary degeneration progressing over several days [23]. In this study, we sought to evaluate neuronal survival one week after reperfusion to be able to detect cumulative damage from both waves of degeneration. Whole retina flatmounts were stained for the neuronal marker NeuN to quantify the number of surviving neurons in the GCL. We observed IR-induced loss of retinal neurons in both WT and Tlr4 KO retinas. However, in contrast to the Tlr4 KO mice, which showed minimal damage (92±2% survival rate), WT mice exhibited significantly (p<0.01, n=6) lower survival of NeuN-positive neurons (63±7%) in the GCL. The NeuN immunohistochemistry showed that the affected neurons were distributed evenly across the ischemic retinas in all treatment groups; no geographic pattern of degeneration was observed (Figure 1A).

### Inactivation of toll-like receptor 4 signaling resulted in reduced inflammation following retinal ischemia

**Figure 2:**
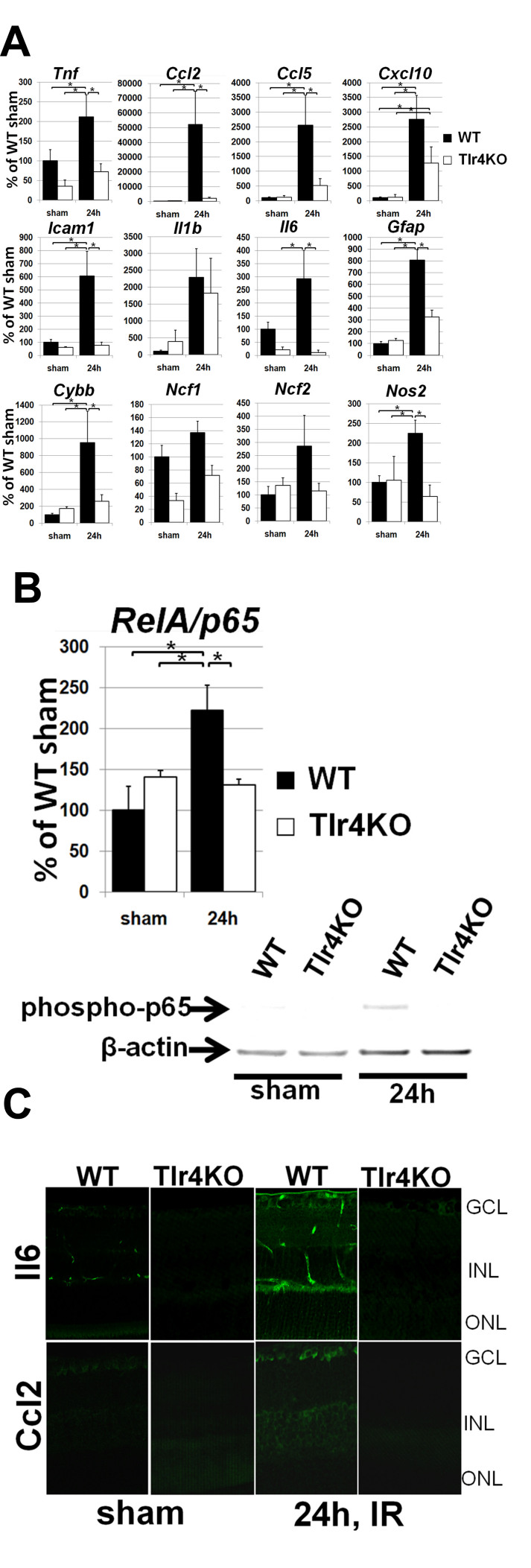
Toll-like receptor 4 deficiency suppresses induction of proinflammatory markers after ischemia/reperfusion. **A**: Differential expression of proinflammatory molecules in the retinas of wild type (WT) and toll-like receptor (Tlr4) knockout (KO) animals 24 h after ischemia/reperfusion (IR). Gene expression was assessed using real time PCR in sham-operated controls and experimental retinas following IR. For each gene, results are expressed as a percentage of corresponding value in the sham-operated eye±SEM after normalization to β-actin. (*p<0.05, n=6). **B**: The levels of p65 (RelA) in Tlr4-deficient mice after retinal IR injury were studied using real time PCR and western blot analysis. **C**: Immunohistochemistry for interleukin 6 (Il6) and chemokine *(*C-C motif*)* ligand 2 (Ccl2) protein accumulation in post-ischemic retinas of WT and Tlr4 KO mice are consistent with increased levels of the transcripts at the level of corresponding proteins. Scale bar equal to 100 μm.

To study the molecular changes associated with the elevated resistance to ischemia in Tlr4 KO retinas, we compared the expression of several proinflammatory genes known to be involved in IR-induced cytotoxicity in experimental versus control eyes. We measured gene expression in total RNA extracted from whole retina 24 h postreperfusion because most changes in gene expression for proinflammatory factors typically occur shortly after IR injury. Transcriptional upregulation of the p65 (*RelA*) subunit of NF-κB, interleukin 1β (*Il1β*), interleukin 6 (*Il6*), tumor necrosis factor (*Thf*), Ccl2, chemokine (C-C motif) ligand 5 (*Ccl5*), chemokine (C-X-C motif) ligand 10 (*Cxcl10*), *Icam1*, nitric oxide synthase 2 (*Nos2*) gene, as well as *Cybb*, neutrophil cytosolic factor 1 (*Ncf1*) and neutrophil cytosolic factor 2 (*Ncf2*), genes encoding subunits of the nicotinamide adenine dinucleotide phosphate (NADPH) oxidase protein complex and glial fibrillary acidic protein (*Gfap*) was evident in all experimental eyes 24 h after reperfusion (Figure 2). In Tlr4 KO mice, however, the expression of *RelA*, *Tnf, Il6, Ccl2*, *Ccl5*, *Cxcl10*, *Gfap*, *Icam1, Cybb,* and *Nos2* was significantly reduced relative to WT. We did not detect statistically significant differences in the IR-induced upregulation of *Il1β, Ncf1,* and *Ncf2* genes between the two genotypes. In addition, the levels of phospho-p65 were significantly increased in WT mice 24 h after IR injury, as demonstrated by western blot (Figure 2B). In contrast, there was no significant difference between the Tlr4 KO sham control and Tlr4 KO IR mice in the levels of phospho-p65 protein. The gene expression profiles for *Il6* and *Ccl2* were consistent with the corresponding protein accumulation levels detected by immunohistochemistry 24 h after reperfusion (Figure 2C). Combined, these data indicate that, as a result of Tlr4 inactivation, the inflammatory response observed in the retina 24 h after reperfusion was significantly suppressed in the retinas of Tlr4-deficient mice.

## Discussion

This study shows that Tlr4-deficient mice have reduced retinal damage and inflammatory response after an IR injury. The neuroprotection observed in Tlr4-deficient mice raises a question concerning which molecular mechanism(s) may be involved. Retinal and brain ischemia results in a prolonged period of neuronal cell death with a high number of necrotic cells at an early stage of pathology [1,2,24–27]. The factors liberated from necrotic cells could trigger inflammation and damage through Tlr4–NF-κB signaling after IR injury [28,29]. Our data indicate that the IR-induced activation of NF-κB was attenuated in the Tlr4 KO animals, which is consistent with previous studies of Tlr4-deficient animals challenged with brain ischemia [12,13]. The NF-κB-regulated expression of *Tnf, Il6, Ccl2*, *Ccl5*, *Cxcl10,* and *Icam1* [22] were significantly reduced in Tlr4 KO versus WT retinas, suggesting that the neuroprotective effect is in part due to Tlr4-NF-κB signaling. A robust inflammatory response in WT animals can exacerbate the injury-induced stress by overexposing neurons to neurotoxic levels of Tnf and Il6 cytokines, as shown previously [30]. The CCL2, CCL5, and CXCL10 chemokines, as well as the cell adhesion molecules ICAM1 are essential for immune cell activation, attraction, and trafficking across the blood-brain barrier into the CNS under both physiologic and pathological conditions [31,32]. Lowered activity of genes encoding these molecules in Tlr4 KO animals suggests that the diminished ability for inflammatory cells to infiltrate the retina could elicit a neuroprotective effect following ischemia. Finally, the excessive activity of the *Cybb* and *Nos2* genes, encoding subunits of the reactive oxygen species (ROS)-producing enzymes NAD(P)H oxidase and inducible NO-synthase, likely causes oxidative stress. The activation of these enzymes is broadly deleterious, and their inhibition was shown to be neuroprotective [33,34]. Our analysis of IR-challenged retinas revealed that the gene expression of *Nos2* and *Cybb* was suppressed in the retina of Tlr4 KO animals relative to WT controls.

In conclusion, our data strongly support the hypothesis that Tlr4 signaling negatively influences neuronal survival and promotes inflammatory gene expression following retinal IR injury. Triggering inflammation and damage through Tlr4 by factors liberated from necrotic cells after IR injury could provide a mechanism of neuroprotection in Tlr4-deficient animals. This mechanism will be further examined in our future studies.
